# PD53 Glucagon-Like Peptide-1 Receptor Agonists And Polyagonists For Severe Obesity: Rapid Overview Of Outcomes Measurement

**DOI:** 10.1017/S0266462325101852

**Published:** 2025-12-29

**Authors:** Thiago Trindade, Maria Eduarda dos Santos Puga, Flávia Tavares Silva Elias

## Abstract

**Introduction:**

Glucagon-like peptide-1 receptor agonist (GLP-1RA/pA) drugs are a breakthrough in obesity management. They produce nearly twice the weight loss achieved by previous drugs and have raised clinical expectations. At least nine GLP-1RA/pA drugs are currently approved or in phase three evaluation. This report aimed to provide an overview of evidence on the clinical outcomes of GLP-1RA/pA drugs to assist topic selection for a complete health technology assessment.

**Methods:**

Exploratory searches were conducted in PubMed, combining the validated database systematic review filter with a structured string for patients with severe obesity (population) and GLP-1RA/pA as the intervention of interest. Inclusion criteria were: (i) systematic reviews with a meta-analysis (SRMA); (ii) severely obese individuals; and (iii) GLP-1RA/pAs as the active intervention. We excluded studies with: (i) children or adolescents only; (ii) any other therapeutic drug class used as an active intervention; (iii) systematic reviews without meta-analyses and other study designs; and (iv) studies in which obesity was not an inclusion criterion.

**Results:**

Forty SRMAs were included. Liraglutide was the most assessed drug, while beinaglutide was the least commonly assessed. Included patients (mean age 12.7 to 75 years) had a mean body mass index of 23 to 34.4 kg/m²; follow-up times ranged from four to 160 weeks. Of the SRMAs, 39 reported efficacy and safety outcomes, while one reported safety outcomes only. Two SRMAs reported other outcomes (economic outcomes and ethnic diversity of clinical trials). Thirty-four SRMAs included randomized controlled trials and five included observational studies that assessed bariatric surgery poor responders (BSPR). Three reviews performed network comparisons. Specific subgroups of obese populations were assessed in eight reviews (BSPR, ethnically diverse people, and obese patients using antipsychotic drugs).

**Conclusions:**

While some reviews have explored specific subgroups, there is a gap regarding severe and super obese patients. Few SRMAs have focused on cardiovascular outcomes. Systematic reviews based on real-world evidence and long-term outcomes are limited. Our findings highlight the need for further research to address these gaps and provide more robust evidence for clinical practice.

